# Supplementary material for: Factors associated with interobserver variation amongst pathologists in the diagnosis of endometrial hyperplasia: A systematic review
Source: PLoS One. 2024 Apr 29;19(4):e0302252. doi: 10.1371/journal.pone.0302252 (PMC11057740; doi:10.1371/journal.pone.0302252)
Supplement: S1 Table — (DOCX) [file pone.0302252.s002.docx]

S1 Table. Intraobserver variation outcomes in the diagnosis of endometrial hyperplasia in two of the included studies.

| **Author and year** | **Intra-observer variation** | |
| --- | --- | --- |
|  | **Agreement (%)** | **Overall *κ*** |
| D’Angelo *et al.,* 2021 | Biopsy diagnosis:  96.2 – 97.5%  Hysterectomy diagnosis:  89.9 – 98.7% | Biopsy diagnosis:  0.91 –0.95  Hysterectomy diagnosis:  0.85 – 0.98 |
| Hecht *et al.,* 2005 | 92.8% | 0.73-0.90 |
