# Supplementary material for: Factors associated with interobserver variation amongst pathologists in the diagnosis of endometrial hyperplasia: A systematic review
Source: PLoS One. 2024 Apr 29;19(4):e0302252. doi: 10.1371/journal.pone.0302252 (PMC11057740; doi:10.1371/journal.pone.0302252)
Supplement: S1 Appendix — (DOCX) [file pone.0302252.s003.docx]

**S1 Appendix. Search strategy for MEDLINE and EMBASE.**

| 1 | exp Endometrial Neoplasms/ or endometrial neoplasm*.mp. |
| --- | --- |
| 2 | exp Uterine Neoplasms/ or uterine neoplasm*.mp. |
| 3 | womb neoplasm*.mp. |
| 4 | gynaecological neoplasm*.mp. |
| 5 | gynecological neoplasm*.mp. |
| 6 | 1 or 2 or 3 or 4 or 5 |
| 7 | exp Endometrial Hyperplasia/ or endometrial hyperplasia*.mp. |
| 8 | simple endometrial hyperplasia.mp. |
| 9 | complex endometrial hyperplasia.mp. |
| 10 | simple atypical endometrial hyperplasia.mp. |
| 11 | complex hyperplasia without atypia.mp. |
| 12 | simple hyperplasia without atypia.mp. |
| 13 | simple hyperplasia with atypia.mp. |
| 14 | endometrial hyperplasia without atypia.mp. |
| 15 | atypical hyperplasia.mp. |
| 16 | non atypical hyperplasia.mp. |
| 17 | atypical endometrial hyperplasia.mp. |
| 18 | non atypical endometrial hyperplasia.mp. |
| 19 | endometrial intraepithelial neoplasia.mp. |
| 20 | EIN.mp. |
| 21 | 7 or 8 or 9 or 10 or 11 or 12 or 13 or 14 or 15 or 16 or 17 or 18 or 19 or 20 |
| 22 | detection*.mp. |
| 23 | diagnosis*.mp. |
| 24 | reporting*.mp. |
| 25 | classification.mp. or exp Classification/ |
| 26 | 22 or 23 or 24 or 25 |
| 27 | "reproducibility of results".mp. or exp "Reproducibility of Results"/ |
| 28 | observer variation.mp. or exp Observer Variation/ |
| 29 | observer agreement*.mp. |
| 30 | observer reliability*.mp. |
| 31 | observer diversity*.mp. |
| 32 | 27 or 28 or 29 or 30 or 31 |
| 33 | pathology*.mp. or exp Pathology/ |
| 34 | pathologists*.mp. or exp Pathologists/ |
| 35 | histopathology*.mp. |
| 36 | histological*.mp. |
| 37 | 33 or 34 or 35 or 36 |
| 38 | 6 and 21 |
| 39 | 26 or 32 |
| 40 | 37 and 39 |
| 41 | 38 and 40 |
| 42 | limit 41 to (humans and yr="2000 -Current") |
| 43 | limit 42 to "review" |
| 44 | 42 not 43 |
