# Supplementary material for: Factors associated with interobserver variation amongst pathologists in the diagnosis of endometrial hyperplasia: A systematic review
Source: PLoS One. 2024 Apr 29;19(4):e0302252. doi: 10.1371/journal.pone.0302252 (PMC11057740; doi:10.1371/journal.pone.0302252)
Supplement: S2 Appendix — (DOCX) [file pone.0302252.s004.docx]

**S2 Appendix. Search strategy for Web of Science.**

| "endometrial neoplasm" or "uterine neoplasm" or "womb neoplasm" or "gynaecological neoplasm" or "gynecological neoplasm" |
| --- |
| **AND** |
| "endometrial hyperplasia" or "simple endometrial hyperplasia" or "complex endometrial hyperplasia" or "simple atypical endometrial hyperplasia" or "complex hyperplasia without atypia" or "simple hyperplasia with atypia" or "simple hyperplasia without atypia" or "simple hyperplasia with atypia" or "endometrial hyperplasia without atypia" or "atypical hyperplasia" or "non atypical hyperplasia" or "atypical endometrial hyperplasia" or "non atypical endometrial hyperplasia" or "endometrial intraepithelial neoplasia" or "EIN" |
| **AND** |
| detection or diagnosis or reporting or classification* |
| **OR** |
| "reproducibility of results" or "observer variation" or "observer reliability" or "observer diversity" |
